# Thoughts on the Psoric Miasm

**Published:** 1892-10

**Authors:** I. Dever

**Affiliations:** Clinton, N. Y.


### THOUGHTS ON THE PSORIC MIASM.

I. Dever, M. D., Clinton, N. Y.

It would be impossible to give more than a passing notice of psora in the necessary limits of this paper, though we hope to present a few facts in relation to this the greatest of chronic miasms.

The discovery of psora as a cause of chronic disease is peculiarly the property of Homœopathy, for so far as we have been able to learn no medical writer anterior to the writings of Hahnemann had hinted at psora as a deep-seated chronic miasm, much less demonstrated the truth of the theory by actual practice.

This psoric theory, as many are pleased to call it, was not a child of the imagination, as they would have us believe, but it was the result of twelve years' painstaking labor and investigation, during which time Hahnemann had put his theory to the severest practical test, and as a result it had borne ripe fruit in the way of cures, long before he announced it as a truth, the understanding and application of which is the only principle that has ever guided the physician to the curative remedy in chronic disease.

When we turn our attention to the life work of Hahnemann we can but regard him as the greatest of medical philosphers, and yet we can have but the slightest glimpse at the enormous amount of thought which he must have expended in clearing away the *débris* of effete and exploded medical systems to give place to his theory of the psoric miasm, which theory he erected on the broad and eternal foundation of truth where it will stand as a monument to his genius as a guide to the treatment of chronic diseases during the lapse of the ages.

Strange as it may appear, all who claim to be homœopathic physicians do not indorse those potent facts so plainly taught in the 79th, 80th, and 81st sections of *The Organon*.

They by their opinions, oppositions, and strange imaginations attempt to substitute a better plan, hence we do not find them treating the cause of disease, but its effect, which leads them into all kinds of by and forbidden paths, until they are hopelessly lost to the art of healing.

Overwhelmed by the mists of polypharmacy, or, what is the same thing, medicines in alternation, they satisfy their conscience by treating an external manifestation of an internal psora by local remedies, ignorantly subjecting their poor confiding patient to some one of the results which are named in section 80 of *The Organon.*

Dr. O. W. Holmes in his Essay on Homœopathy and other kindred "delusions" dismisses the subject of psora in these words: "It entered into my original plan to treat of the doctrine of psora or itch—an almost insane conception which I am glad to get rid of, for this is a subject one does not care to handle without gloves." Such disposition of a profound and important subject is hardly worthy the reputation of so learned a man. But it is a happy illustration of the words of the greatest of teachers, "They have ears but they hear not," "They have eyes but they see not."

He who would touch the sympathetic chord and cause it to vibrate in perfect harmony must do it with an ungloved hand; and those who would comprehend the doctrine of psora as taught by Hahnemann and his followers must approach the subject with a clear head and an honest heart.

Ever since the morning on which Adam learned the external application of fig leaves, it has been appointed unto man to eat his bread by the sweat of his brow. On the other hand, it is no compliment to our knowledge of a profound subject to dismiss it without an investigation as to whether it is true or false, and these conclusions cannot be definitely settled in the minds of those who have not arrived at mature judgment through the "sweat of the brain."

I do not think there are many who would have anything like a correct idea of psora by reading sections 79, 80, and 81 the first time. But what physician who has given those sections of *The Organon* careful and patient thought, standing in the light of experience, will say that the doctrine of psora as taught in the above-named sections is a delusion and has no foundation in fact? The opponents of Homœopathy and even some who are favorably inclined to the law of cure, insist that psora and the itch are synonymous, but, unless I am mistaken, *The Organon* does not so teach; but it does teach that psora is the result of scabies or itch, which left uncured may be and often is transmitted from generation to generation and in this way becomes more potent from having passed through the systems of so many different individuals. This is a fact in harmony with the law of potentization, and while observation teaches that scabies in its primary stage is not a difficult disease to cure, psora in its different forms may severely tax the skill of the physician, and when complicated with either of the other chronic miasms (syphilis or sycosis) it may defeat his best efforts. See section 206 *Organon*, which teaches the possibility of a complication of the above-named miasms.

But it is the secondary symptoms of psora with which we as homœopathic physicians have to deal—some deep-seated dyscrasia—handed down from our ancestors from generation to generation in its numerous forms with its multitude of symptoms, presenting every phase of acute and chronic difficulty known to the catalogue of diseases. From a good experience in the treatment of both acute and chronic diseases, I am more than satisfied that if it were possible to eradicate the psoric miasm from the human family that sickness would be reduced to the minimum, and barring accident, man would live to a painless, happy old age and fall quietly to sleep in death.

The fact that psora is a potent factor in all diseases, both acute and chronic, has not escaped the observation of a large number of physicians who have had an experience in the treatment of the so-called zymotic diseases as small-pox, scarlet fever, and measles, from which object lesson they have learned that the malady will be mild or severe in ratio to the individual psoric condition of the patient.

In conclusion, it may not be amiss to say that though this scientific discovery cost Hahnemann years of patient investigation and thought, he so thoroughly simplified and demonstrated it as the only principle which accounts for a large majority of human ills that a failure on the part of the physician to investigate the doctrine of psora in all of its details cannot be regarded as a neglect of duty only, but in the light of the success which has ever attended its true and faithful adherents, such neglect becomes a crime.

Section 80 of *The Organon* is replete with practical suggestions which shine out bright as a beacon light to guide the homœopathic craft clear of the rocks and shoals of empiricism.

### Discussion.

Dr. Tompkins—I had always supposed that the *suppression* of scabies was what induced the psoric diathesis. I do not recall Hahnemann's teaching in regard to whether psora may result from a spontaneous disappearance of that disease.

Dr. A. G. Allan—I understand that the disastrous consequences of psora are due to the suppression of a local disease by local treatment, driving the disease from its superficial local seat to more important internal structures.

The treatment of the allopaths drives the disease from one organ to another, until finally it reaches the internal and vital parts of the body, when the patient becomes a total wreck. We can cure the itch with internal homœopathic medicines and never see the dangerous results which follow the use of Sulphur ointment.

Dr. J. H. Allen—The itch is not a local or superficial disease, but a deep, internal disorder, and the eruption is the expression of it.

Dr. A. G. Allan—No eruption ever occurs in a disease unless the organism has been brought under the influence of a miasm. Then the eruption appears. Chancre never occurs until the system has been brought under the influence of the poison, and the reason we get bad results when we suppress chancres and psoric manifestations is because we prevent the miasm from expressing itself in its simple form upon the skin.

Dr. A. R. Morgan—I entirely dissent from the idea that Hahnemann attributed psora to the acarus parasite. The term psora was at that day applied to nearly all cutaneous eruptions. I think that the itch insect was known to Hahnemann. The psora of Hahnemann is not the itch, but the itch was the result of implanting a peculiar virus upon an organism tainted by psora.

Dr. Davis—I had a case of suppressed itch, in which an acarus was found in the urine. The itch had been suppressed fifteen or twenty years before.

Dr. C. E. Hastings—This urine I examined. I attributed the presence of the acarus to accidental causes. The urine was perfectly normal. I asked the doctor if his patient was troubled with scabies, and was told that years before he had had it and it was suppressed.

Dr. Kennedy—How many physicians present have treated scabies homœopathically and succeeded in holding their patients until cured?

Several Voices—I have.

Dr. Fincke—I differ from Dr. Morgan's statement. When Hahnemann taught the psora doctrine, he knew nothing of the insect. Itch was known simply as an itching, vesicular eruption on the skin, and it was long after that time that the acarus was discovered. The itch does not exactly depend upon the acarus, but upon the internal miasm which gives the acarus a soil to live upon. This is shown by the experiments of certain students, who infected themselves with the acarus itch. Not all of them got the acarus, but all of them got the itch.

Dr. Sawyer—There is plenty of itch in Indiana; we have all had it down there; some of us several times. I have had thousands of cases of it to treat, and I hold them until they are cured with straight Homœopathy and high potencies. I find the acarus in some cases but not in all.

Dr. A. R. Morgan—I have been in the habit of applying lard to kill the parasite, at the same time giving the appropriate remedy.

Dr. Tompkins—I have never cured the itch with high potencies, but I have cured a number of cases with the tincture of Sulphur used two or three times a day for a considerable time, using no external application at all.

Dr. Sawyer—I would suggest that perhaps the doctor's cases of itch were suppressed, and not cured.

Dr. Reed—In cases of scabies we have an insect, the acarus, which can easily be seen traveling over the skin. Now, what are you going to do with that little animal? Can you kill him with the simillimum?

Voices—Yes, yes!

Dr. A. G. Allan—In New Jersey there are mosquitoes of large and robust build, of a ferocious nature, and insatiable appetite. Now, the homœopaths there give high potencies of mosquito (culex) to prevent the people from being bitten by the animals. If that can be done with mosquitoes, why not the same with the acarus?

Dr. McLaren—In regard to the acarus, it only differs in degree from lice, which collect and find a suitable ground or soil in an eczemetous skin, and which have been cured by Psorinum without any external application whatever. I should like to suggest to Dr. Morgan that it is an open question whether the lard kills the acarus, or whether it simply dies from the action of the simillimum. The curse pronounced in Eden, "in the sweat of thy face shalt thou eat bread," finds a remarkable fulfillment in the various manifestations of psora which are often greatly relieved by perspiration—a strong indication for Psorinum.

Dr. Reed—What are you going to do with the proposition of Hahnemann, when he says, take a little of the oil of Lavender and touch that insect and it will kill it every time?

Dr. Fincke—This about the oil of Lavender must come from some of Hahnemann's earlier writings. It is impossible that he recommended it to kill the acarus, for the acarus was not discovered until after his time. Let me call to your mind that by means of our high potencies we are able to kill and expel much larger animals than the itch insect. Do we not expel tapeworms and other large worms? Dr. Baylies reported a case of tape-worm expelled by a dose of Lycopodium$^{M}$ (F.).

Dr. Long—I have never had a case of itch to treat that I know of, but I have had two cases of maggots in superficial ulcers cured by the remedy, which was Arsenic, within three days.

Dr. I. Dever—I believe that the insect is not the cause of scabies, but the result; just as I believe that bacteria are the result and not the cause of disease. Hahnemann teaches that psora is a transmissible disease, and that it becomes more potent as it passes through many people and generations.

